# Validating Wing Biopsies for Blood‐Borne Pathogen Characterization in Bats

**DOI:** 10.1111/mec.70560

**Published:** 2026-09-29

**Authors:** Molly C. Simonis, Amanda Vicente‐Santos, Lauren R. Lock, Kristin E. Dyer, Beckett L. Olbrys, M. Brock Fenton, Karen E. Sears, Dmitriy V. Volokhov, Nancy B. Simmons, Daniel J. Becker

**Affiliations:** ^1^ School of Biological Sciences University of Oklahoma Norman Oklahoma USA; ^2^ College of Forestry, Wildlife and Environment and College of Veterinary Medicine Department of Pathobiology Auburn University Auburn Alabama USA; ^3^ Department of Biology University of Western Ontario Ontario Canada; ^4^ Department of Ecology and Evolutionary Biology University of California Los Angeles Los Angeles California USA; ^5^ Center for Biologics Evaluation and Research, U.S. Food and Drug Administration Silver Spring Maryland USA; ^6^ Department of Mammalogy, Division of Vertebrate Zoology American Museum of Natural History New York New York USA

**Keywords:** bartonellae, hemoplasmas, trypanosomes, vampire bat

## Abstract

Wildlife surveillance is critical for tracking disease emergence, characterizing pathogen diversity, and assessing spillover risks. Blood‐borne pathogens are of particular interest for such efforts due to their global distribution, broad host taxa, and zoonotic potential. Despite the need to monitor blood‐borne pathogens, blood collection is costly for biologists and sampled wildlife (i.e., time‐consuming and stressful), hindering our ability to expand surveillance efforts. Using vascular tissues to detect infections can minimize sampling effort and stress on wildlife, but it is unclear how pathogen detection and characterization from these tissues compared to blood. To evaluate the reliability of using vascular tissues for detecting blood‐borne pathogens in wildlife, we collected paired samples of blood and wing biopsies from common vampire bats ( *Desmodus rotundus* ) and molecularly screened for bartonellae, hemotropic mycoplasmas (hemoplasmas), and trypanosomes. Probabilities of detection were lower in wing tissues than in blood for all pathogens, possibly due to blood vessel avoidance when collecting the former. However, we detected infection in wing tissues of at least two individual bats for each pathogen. Paired‐positive individuals mostly showed high sequence concordance between tissues, and sample sizes needed to detect a single infection and observed prevalences were similar (i.e., *n* = 10–39). Due to lower probabilities of infection in wing tissues compared to blood, we suggest that using these samples to estimate infection prevalence of blood‐borne pathogens is not ideal. However, our results demonstrate that vascular tissues can be used for initial pathogen assessment and discovery to help target surveillance efforts in the future.

## 1 Introduction

As emerging infectious diseases threaten both wildlife and human health (Jones et al. 2008; Daszak 2000), surveillance of wildlife pathogens is a critical aspect of tracking disease emergence, particularly zoonotic pathogens (Sharan et al. 2023; Watsa and Wildlife Disease Surveillance Focus Group 2020). Characterizing wildlife pathogens for surveillance is important for managing and predicting disease outbreaks, epidemics, and pandemics (Artois et al. 2009), but there are many challenges associated with wildlife sampling for pathogen detection (Ryser‐Degiorgis 2013; Delgado et al. 2023). For example, efforts to survey wildlife can be costly in terms of time and effort associated with animal capture (particularly for cryptic taxa), and invasive sampling strategies (e.g., blood collection) can cause additional stress to wildlife. Therefore, methods for field sampling and pathogen detection that reduce researcher effort and wildlife stress are important for sustainable, ongoing surveillance of zoonotic pathogens.

Blood‐borne bacterial and protozoan pathogens are zoonoses of particular and growing concern (Boulouis et al. 2005; Millán et al. 2021; Colwell et al. 2011), as they are geographically widespread across wildlife taxa (Becker et al. 2024; Volokhov et al. 2023; Kang et al. 2013; André et al. 2011; Álvarez‐Fernández et al. 2018; Fisher et al. 2025; Bergner et al. 2021; Martínez‐Hernández et al. 2022; Ansil et al. 2025). Bacterial bartonellae and hemotropic mycoplasmas (hemoplasmas), as well as protozoan trypanosomes, have varying routes of transmission. For example, bartonellae in bats have evidence of being largely vectored by arthropods (McKee et al. 2026), but *Bartonella henselae* , which causes cat scratch fever, is typically directly transmitted to humans through cat bites and scratches (although vectored transmission is still possible (Breitschwerdt 2008)). Hemoplasmas show evidence of direct transmission between bats and humans (via bat consumption (Descloux et al. 2021; Klement‐Frutos et al. 2023)), vertical transmission within small mammalian host species (Millán et al. 2021), and potential vector transmission (Willi et al. 2007). Trypanosomes are commonly vector‐transmitted via triatomine urine and faeces and biting fly saliva as well as through direct oral transmission (de Noya and González 2015; Desquesnes et al. 2013). Due to varying transmission routes, hosts, and evidence of cross‐species transmission, we need surveillance of these blood‐borne bacterial and protozoan pathogens to better understand their genetic diversity in wildlife and spillover risks.

Surveillance of blood‐borne pathogens in wildlife typically involves detecting infections from blood samples, but collecting blood from wildlife can be time‐consuming, require specialized training, and can place significant stress on individuals being sampled (Widmaier and Kunz 1993; Pavlova et al. 2018; Arnold et al. 2008). Blood‐borne bacterial and trypanosome infections can be detected in highly vascularized organ tissues such as the spleen, liver, and heart (Lima et al. 2025; Trüeb et al. 2018; Szentivanyi et al. 2023). However, collecting organs can be time‐intensive and requires lethal sampling, which may run counter to conservation concerns, conflict with other ongoing wildlife studies, and present permitting issues.

Vascular tissue sampling methods for blood‐borne pathogens that are logistically easier, less time‐consuming, non‐lethal, and minimally invasive, such as earclips from domestic cats and cattle (Manvell et al. 2021; Quinet et al. 2016), ear biopsies and toe clips in rodents (Beckmann et al. 2019; Sinsky and Piesman 1989) or wing tissues in bats (Nichols et al. 2019; Lourenço et al. 2018; Quiroga et al. 2022), may contain enough blood to detect infections and also minimize sampling effort and stress on individual wildlife. Unlike blood samples, tissue samples such as these are routinely collected and vouchered in museums (Thompson et al. 2021; Cronin et al. 2025), many of which are increasingly stored cryogenically (Cronin et al. 2025), so tissues may be accessible without sampling new individuals in the field. However, it is unclear how low‐effort vascular tissue sampling compares with direct blood collection for our ability to detect and identify blood‐borne pathogens.

Bats are commonly associated with emerging zoonoses, including blood‐borne pathogens such as bartonellae, hemoplasmas, and trypanosomes (Becker et al. 2024; Descloux et al. 2021; Klement‐Frutos et al. 2023; Lourenço et al. 2018; Lilley et al. 2017; Ramírez et al. 2014). Bat wings are highly vascularized, and wing biopsies are commonly collected for molecular species identification (Walker et al. 2016), stable isotope analyses (Roswag et al. 2014), characterizing population structure (Grimshaw et al. 2024), quantifying aging (Lu et al. 2023; Haghani et al. 2023; Wilkinson et al. 2021) and ‐omics studies of species diversity (Kruskop et al. 2012). While bat wing biopsy samples have been used to detect blood‐borne pathogens previously (Nichols et al. 2019; Quiroga et al. 2022), it is unclear how using wing tissues instead of blood, or sampled complementarily with blood, could facilitate surveillance.

Here, we use paired blood and wing biopsy samples from common vampire bats ( *Desmodus rotundus* ) collected in Belize to determine concordant infection detection across the two sample types. We used blood and wing tissues from each individual to investigate detection probability for infections with bartonellae, hemoplasmas, and trypanosomes, all of which are known to occur with high prevalence in the local vampire bat population (Volokhov et al. 2023, 2017; Becker et al. 2021; Dunsmore et al. 2025; Torgerson et al. 2026; Lock et al. 2026). We hypothesized that the probability of detecting infection would be lower in wing biopsy samples compared to blood due to collection methods (i.e., researchers typically avoid visible wing blood vessels when collecting biopsies) and blood volumes in those samples compared to whole blood.

## 2 Methods

### 2.1 Vampire Bat Sampling

During two, two‐week trips in November 2021 and April–May 2022, we sampled common vampire bats in Orange Walk District, Belize as part of a long‐term study of the local bat community (Volokhov et al. 2023; Lock et al. 2026; Becker et al. 2025). Bats were captured using mist nets and harp traps along flight paths within the Lamanai Archeological Reserve and Ka'Kabish Archaeological Site (Lock et al. 2026; Herrera et al. 2018; Ingala et al. 2019), and identified by their distinct morphological features in the field upon capture using taxonomic keys (Medellín et al. 2007; York et al. 2019; Reid 2009). Bats were marked with 3.5 mm incoloy wing bands (Porzana Inc.), and we obtained blood by lancing the propatagial vein with 23‐gauge needles, followed by collection in heparinized capillary tubes. Blood was stored on Whatman FTA cards and at ambient temperature in the field prior to long‐term preservation at −20°C at the University of Oklahoma. We also collected two 2 mm wing biopsy punches along the lower wing membrane close to the body wall and stored them in 1 mL DNA/RNA Shield at −80°C. All bats were released following sampling.

Field procedures were performed according to guidelines for the safe and humane handling of bats published by the American Society of Mammalogists (Sikes and the Animal Care and Use Committee of the American Society of Mammalogists 2016) and were approved by the Institutional Animal Care and Use Committees of the American Museum of Natural History (AMNH IACUC‐20210614) and the University of Oklahoma (2022–0197). All bat sampling was authorized by the Belize Forest Department through scientific collection permit FD/WL/1/21(12) and Belize Institute of Archaeology permits IA/S/5/6/2l(01) and IA/H/1/22(03).

### 2.2 DNA Extraction

As described previously, we extracted genomic DNA from 4–5 2 mm punches of blood from Whatman FTA cards using QIAamp DNA Investigator Kits (Qiagen) following the manufacturer's instructions (Volokhov et al. 2023; Lock et al. 2026; Becker et al. 2025). From a broader collection of blood samples, we here focused analyses on individual bats with paired blood and wing biopsies (*n* = 63). Limited availability of raw sample on FTA cards enabled screening of 60 bats for bartonellae, 60 bats for trypanosomes, and 62 bats for hemoplasmas. Due to ongoing projects across institutions using wing tissues, we used two different DNA extraction methods. Most wing biopsy samples (*n* = 45) were processed at University of Oklahoma using the *Quick*‐DNA/RNA Viral Magbead Kit (Zymo Research) run on an IsoPure 96 platform (Accuris Instruments). Briefly, we used a vortex mixer to homogenize wing biopsy samples (one biopsy punch per individual) and included a 15‐min proteinase K digestion at room temperature per the manufacturer's instructions. DNA was eluted into 50 μL nuclease‐free water. The remaining wing biopsy samples (*n* = 18; one biopsy punch per individual) were processed at University of California Los Angeles using the *Quick*‐DNA Miniprep Plus Kit (Zymo Research), following the manufacturer's solid tissue protocol. Prior to extraction, we used a rotor‐stator homogenizer to homogenize wing biopsy samples (one biopsy punch) followed by an overnight proteinase K digestion at 55°C. DNA was eluted into 30 μL nuclease‐free water. DNA extraction methods for wing biopsy samples yielded 0.02 ng/μL greater concentrations (measured via an Invitrogen Qubit Fluorometer, ThermoFisher Scientific) when using the *Quick*‐DNA Miniprep Plus Kit compared to the *Quick*‐DNA/RNA Viral Magbead Kit (generalized linear model [GLM] with Tweedie response: *F*_(1)_ = 40.1, *p* < 0.0001, *R* ^2^ = 0.47). However, DNA purity (260/280) did not differ between extraction methods (GLM with Tweedie response: *F*_(1)_ = 0.62, *p* = 0.43, *R* ^2^ = 0). Finally, DNA concentrations were 0.11 ng/μL greater in blood than wing tissue when analysing paired samples (generalized linear mixed model [GLMM] with Tweedie response and random effect of individual bat: *F*_(1, 63)_ = 12.94, *p* < 0.001; *R* ^2^ = 0.40), but DNA purity did not differ between paired samples (GLMM with the same structure: *F*_(1, 59)_ = 2.1, *p* = 0.15, *R* ^2^ = 0.57).

### 2.3 Pathogen Screening

We used previously established protocols to screen all paired blood and wing tissue DNA samples for bartonellae, hemoplasmas, and trypanosomes (Table S1). For bartonellae and trypanosomes, we used nested PCR to target the *gltA* (340 bps) and small subunit rRNA (SSU rRNA) genes (650 bps), respectively (Noyes et al. 1999; Birtles and Raoult 1996; Norman et al. 1995). While the former PCR is highly specific to bartonellae, the latter PCR amplifies all trypanosomatids except salivary trypanosomes, unlike methods targeting *Trypanosoma cruzi* specifically (Vicente‐Santos et al. 2023). Positive controls used were *Bartonella doshiae* , *Trypanosoma cruzi*, and *Candidatus Mycoplasma haematozalophi*. Nuclease‐free water was used for all negative PCR controls. Amplicons were purified with Zymo kits (DNA Clean and Concentrator‐5, Zymoclean Gel DNA Recovery) and directly Sanger sequenced at the North Carolina State University Genomic Sciences Laboratory. For hemoplasmas, we used PCR targeting the 16S rRNA gene (Becker et al. 2024; Volokhov et al. 2023, 2017; Lock et al. 2026), with amplicons purified and directly Sanger sequenced at Psomagen. Forward and reverse sequences were trimmed and cleaned in Geneious (Kearse et al. 2012), and we used NCBI BLASTn and previous protocols to assign consensus sequences to genotypes (Becker et al. 2024, 2018, 2020; Lock et al. 2026). Data for bartonellae and hemoplasmas in blood have been published previously (Lock et al. 2026), whereas trypanosome data for blood and data for all pathogens in wing tissues were generated here. Infection positivity in wing tissues did not differ by extraction methods for bartonellae, but was greater for hemoplasmas using wing tissues extracted via the *Quick*‐DNA/RNA Viral Magbead Kit compared to the *Quick*‐DNA Miniprep Plus Kit (GLM with Tweedie response for each pathogen; Table S2). There was also an effect of extraction method on trypanosome infection positivity, but a post hoc pairwise contrast indicated no difference (GLM with Tweedie response; Table S2). As such, we controlled for these differences in our subsequent analyses of paired blood and wing tissues (see *Statistical analyses* below).

### 2.4 Statistical Analyses

All statistical analyses and data visualizations were performed in the statistical environment R (version 4.3.1, Beagle Scouts) and the package *ggplot2* (R Core Team 2023; Wickham 2016). To determine how infection detection differed between blood and wing tissues, we fit binomial GLMMs using the package *lme4* (Bates et al. 2015) for infection status of each pathogen as a function of sample type and a random intercept of individual bat to account for multiple measures (each individual had blood and wing tissues sampled). Given the above differences in pathogen detection for wing tissues extracted using different kits, we also included a random effect of extraction method in our analyses (Oberpriller et al. 2022). We tested each model via a type‐II ANOVA from the *car* package (Fox and Weisberg 2019), and we quantified post hoc estimated marginal means and confidence intervals using the *emmeans* package (Lenth 2021). We present all GLMM coefficients exponentiated as odds ratios (ORs).

Finally, to determine the sample size needed to detect infections in wing tissues, we calculated binomial probabilities using a 95% confidence level and 80% statistical power. We first estimated the minimum sample size for detecting a single infection in blood and wing tissues, such that the probability of zero detections was denoted by (1−*p*)^*n*^, and the probability of a single detection was denoted by 1−(1−*p*)^*n*^, where *p* is the resulting infection probability of bartonella, hemoplasma, or trypanosome infections found here. We then also estimated the sample size needed to detect the observed prevalence of bacterial and protozoan pathogens found in wing tissues only using the *pwr* package in R (Champely 2020), again assuming a 95% confidence level in detection probability and 80% statistical power.

## 3 Results

Of the 63 bats screened (60 for bartonellae, 60 for trypanosomes, and 62 for hemoplasmas), 49 were positive for bartonellae (mean [lower CI, upper CI]; 82% [70%, 89%]; 48 positive from blood, three positive from wing tissues), 38 were positive for hemoplasmas (61% [49%, 72%]; 33 positive from blood, nine positive from wing tissues), and 26 were positive for trypanosomes (43% [32%, 56%]; 24 positive from blood, two positive from wing tissues). Two individuals were positive for bartonellae in both blood and wing tissues, and five were positive for hemoplasmas in both tissues (Table 1). One, four, and one bat were positive in wing tissues but not blood for bartonellae, hemoplasmas, and trypanosomes, respectively. Fourteen bats were coinfected with all three pathogens, two of which would not have been detected without wing tissue screening. One individual was coinfected with bartonellae and trypanosomes only. For individuals that tested positive in both blood and wing tissue, bartonellae sequences were 100% similar between blood and wing tissues, whereas hemoplasma sequences were 98.66% ± 0.54% (mean ± SE) similar between samples (Table 1).

**TABLE 1:** Percent similarity between pathogen sequences identified in all individual vampire bats with paired infection positivity (i.e., infections found in both blood and wing tissues).

BZID	Pathogen	Blood accession and identity	Wing accession and identity	Percent similarity^a^	Lineage match
123	Bartonellae	PQ758887; DR11 genotype	PV599394; DR11 genotype	100	Y
1435	Bartonellae	PQ758926; DR1 genotype	PV599395; DR1 genotype	100	Y
138	Hemoplasma	OQ546566; VB2 genotype	PX705794; VB2 genotype	99.7	Y
1598	Hemoplasma	OQ385172; VB1 genotype	PX705791; VB2 genotype	97.2	N
372	Hemoplasma	OQ546522; VB1 genotype	PX705793; VB1 genotype	99.8	Y
79	Hemoplasma	OQ546567; VB3 genotype	PX705797; VB3 genotype	99.0	Y
1042	Hemoplasma	OQ385170; VB1 genotype	PX705792; VB2 genotype	97.6	N

*Note:* BZID represents the unique identifier for each individual bat. Lineage match indicates if paired infections had the same genotype; genotypes are characterized in more detail elsewhere (Volokhov et al. 2017; Lock et al. 2026; Becker et al. 2018). While the study detected trypanosome infections in wing tissues from two individuals, no cases showed paired positivity (infection detected in both blood and wing tissues from the same individual), therefore no trypanosome sequences are included in this genetic concordance analysis.

^a^ Genetic similarity between blood and wing tissue sequences.

The probability of detecting infection was consistently lower in wing tissues than in blood samples across all three pathogens. However, despite lower detection probabilities for wing tissues compared to blood, we did detect at least two positives in wing tissue for each pathogen. Detecting infections of bartonellae in wing tissues (0.05 [0.02, 0.14]) were 98% less likely than in blood (0.77 [0.65, 0.86]; OR = 0.02, *p* < 0.0001, *R* ^2^_m_ = 0.57, *R* ^2^_c_ = 0.57). Detecting hemoplasma infections in wing tissues (0.15 [0.05, 0.38]) were 92% less likely than in blood (0.69 [0.38, 0.89]; OR = 0.08; *p* < 0.0001, *R* ^2^_m_ = 0.28, *R* ^2^_c_ = 0.41). Finally, detecting trypanosome infections in wing tissues (0.03 [0.01, 0.12]) were 95% less likely than those in blood (0.39 [0.27, 0.51]; OR = 0.05, *p* = 0.0001, *R* ^2^_m_ = 0.40, *R* ^2^_c_ = 0.40; Figure 1).

**FIGURE 1:**
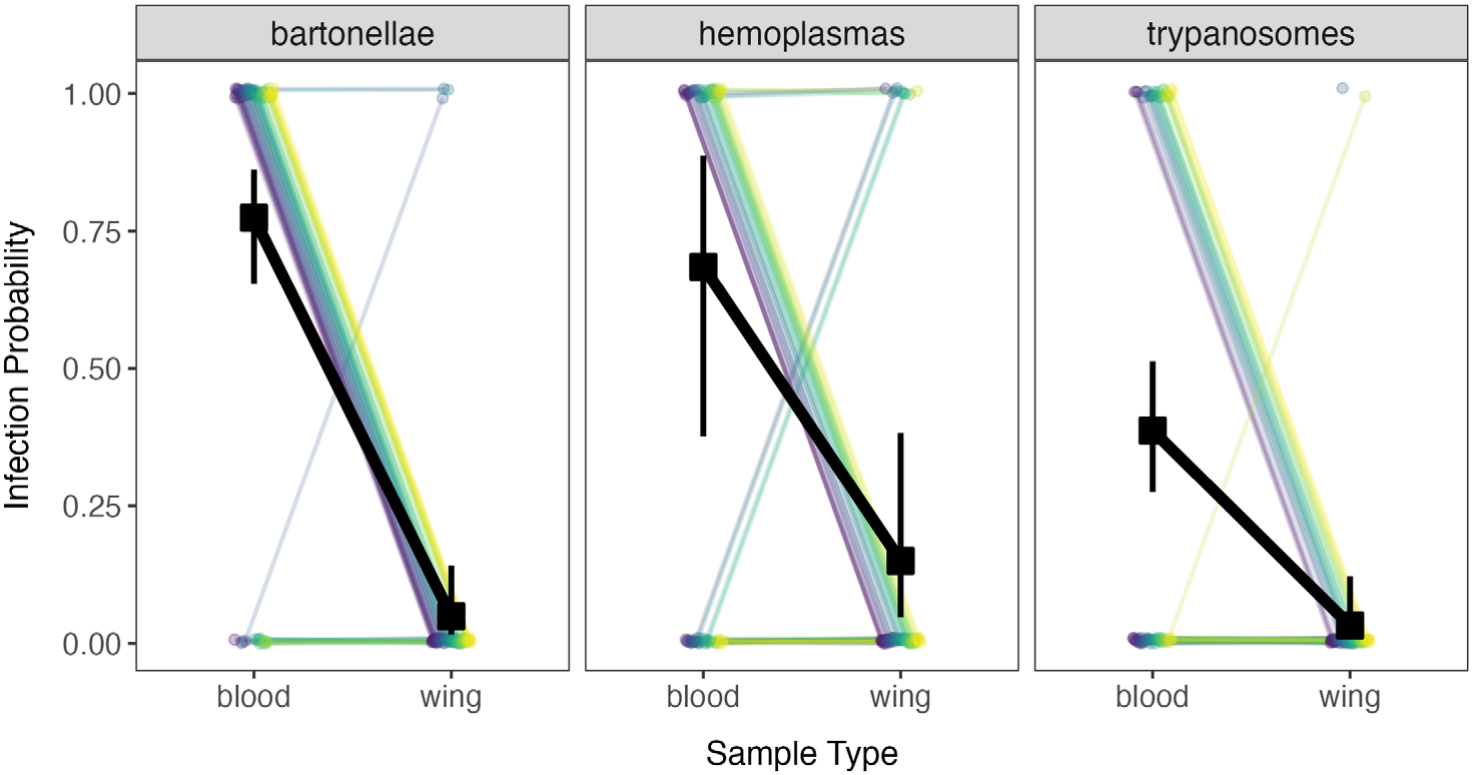
Probability of infection between blood and wing tissues in vampire bats when screened for bartonellae (left; *n* = 60), hemoplasmas (middle; *n* = 62), and trypanosomes (right; *n* = 60). Coloured points and lines represent paired detection results for each individual bat. Black points, bars and lines represent mean infection probabilities and 95% confidence intervals from the respective GLMMs across sample types.

The minimum estimated sample size to detect a single infection in wing tissues with 80% statistical power and 95% confidence was 31, 10, and 32 for bartonellae, hemoplasmas, and trypanosomes, respectively (Figure 2). Comparatively, the minimum estimated sample size to detect a single infection in blood with 80% statistical power and 95% confidence was one, two, and three for bartonellae, hemoplasmas, and trypanosomes, respectively (Figure 2). The minimum estimated sample size to obtain an expected prevalence of 5% (bartonellae), 15% (hemoplasmas), or 5% (trypanosomes) with 80% statistical power and 95% confidence was 39, 13 and 39, respectively (Figure 2).

**FIGURE 2:**
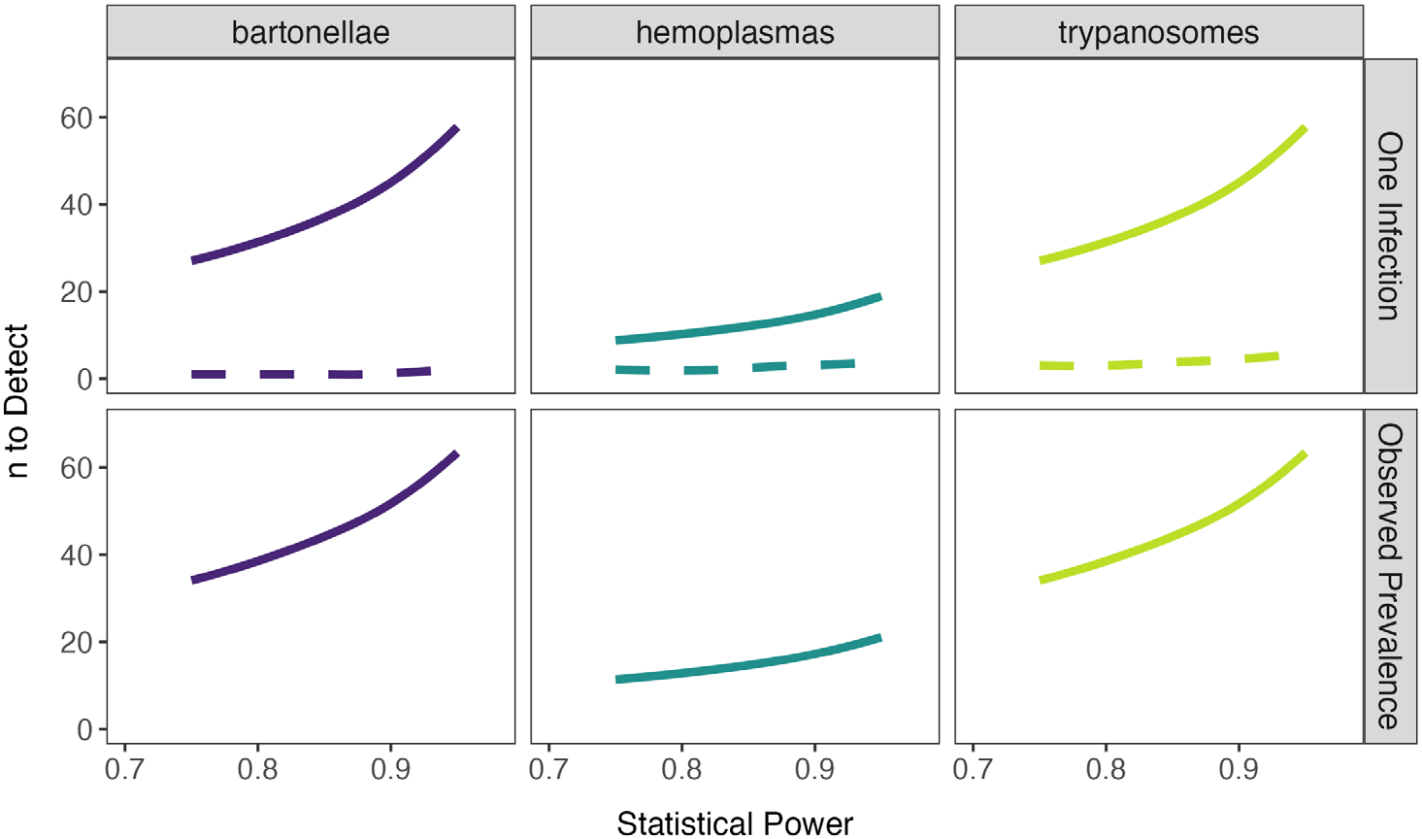
Estimated sample sizes needed to detect a single infection (top) and observed prevalence (bottom) for bartonellae, hemoplasmas and trypanosomes detected in wing tissues. Dashed lines in the top row represent the comparative number of blood samples needed to detect just one infection. Note that axes differ between rows.

## 4 Discussion

Pathogen discovery and surveillance are critical for predicting and managing emerging infectious diseases. Capturing and sampling wildlife can be time and labour intensive as well as stressful for study animals, so identifying field methods that minimize researcher sampling effort and wildlife stress is key to efficient and humane pathogen monitoring. Here, we detected blood‐borne pathogens in vampire bats using paired blood and wing tissue samples. Our results demonstrate that wing tissues represent a biologically relevant sample type for pathogens that require less researcher effort and physiological stress to bats than does blood collection.

The odds of detecting blood‐borne pathogens in wing biopsy samples were substantially lower than blood samples, with the likelihood of detecting these infections being 98%, 92%, and 95% less likely for bartonellae, hemoplasmas, and trypanosomes, respectively. While we expected this reduced detectability given that researchers typically avoid blood vessels when collecting wing biopsies in bats, we show here that wing tissues are a complementary option for initial pathogen detection and genetic characterization. However, it is important to note that six infections would not have been detected without wing tissue screening (i.e., one infection with bartonellae, four hemoplasma infections, and one trypanosome infection), but we would suggest this is likely due to blood within those tissues and not tropism within the wing tissue itself. Our detection probabilities are comparable to those reported in other studies that used wing tissues for detecting blood‐borne pathogens. For example, trypanosomes from wing tissues were detected in four of 17 *Desmodus rotundus* in Brazil (Lima et al. 2025) and one of 361 wing tissues from *Tadarida brasiliensis* (Mexican free‐tailed bats) in Oklahoma, USA (Nichols et al. 2019), whereas we detected trypanosomes in three of 60 wing tissues. While we found that infection probabilities were consistently higher in blood samples than in wing tissues, all three pathogens were detectable to some degree in this sample type. DNA sequences from pathogens identified in wing tissues typically matched those from blood in the same individual bat, indicating the same infections were detected. We therefore suggest that wing tissues could be suitable for initial pathogen screening assessments and help guide further surveillance efforts. Despite the lower prevalence in wing tissues, such tissues are especially promising for blood‐borne pathogen surveys of bats, given that they are easy to collect in large numbers and are commonly collected in field surveys.

Using bat wing tissues for detecting blood‐borne pathogens could be applied more widely in pathogen discovery efforts. Given that wing tissues are often collected for use in molecular species identification (Walker et al. 2016), stable isotope analyses (Roswag et al. 2014), characterizing population structure (Grimshaw et al. 2024), quantifying aging (Lu et al. 2023; Haghani et al. 2023; Wilkinson et al. 2021), and ‐omics studies of species diversity (Kruskop et al. 2012), there are a vast number of wing tissue samples stored across the bat research community. Similarly, wing tissues are commonly collected during routine sampling expeditions for museum collections. Future studies could compare infection detection in wing tissues across multiple storage methods (e.g., ethanol, formalin‐fixed, silica beads, DNA/RNA Shield) to ensure successful application for pathogen discovery more broadly. These samples could be used for initial assessments to determine which blood‐borne pathogens may be circulating. Researchers could then decide whether additional efforts are needed for targeted surveillance (e.g., longitudinal study), using blood collection to obtain more accurate estimates of infection prevalence and better characterize genetic variants and determine zoonotic risks.

We used two different but methodologically similar approaches to extract DNA from wing tissues. DNA concentrations were, on average, 0.02 ng/μL greater when extracted using the *Quick*‐DNA Miniprep Plus Kit compared to the *Quick*‐DNA/RNA Viral Magbead Kit. We suggest this difference in concentrations is negligible, given that DNA purity did not differ between extraction methods and infection positivity did not differ between methods for both bartonellae and trypanosome infections. Although wing tissues extracted using the *Quick*‐DNA/RNA Viral Magbead Kit were 86% more likely to be positive for hemoplasmas than those extracted using the *Quick*‐DNA Miniprep Plus Kit, we suggest this difference is more likely due to the sample size imbalance between kits used and the general rarity of hemoplasma infections in wing tissues. Therefore, blood‐borne infection testing from wing tissues is likely reliable regardless of extraction methods used.

We were able to detect infections of bartonellae, hemoplasmas, and trypanosomes in at least two vampire bats for each pathogen. Six infections would not have been detected without wing tissue screening (one infection with bartonellae, four hemoplasma infections, and one trypanosome infection). However, our ability to detect infections in wing tissue varied by pathogen (i.e., three infections with bartonellae, nine hemoplasma infections, and two trypanosome infections), and achievement of genetic concordance in individuals with paired blood and wing infections was limited to bartonellae only. Limitations in wing tissue detections and genetic concordance could be due to the tropism spectrum of each pathogen. For example, bartonellae typically infect red blood cells and endothelial walls of blood vessels (Eicher and Dehio 2012; Dehio 2004), and hemoplasmas are intracellular infections of red blood cells (although they can also infect reproductive tissues) (Messick 2004). Trypanosomes are known for varied tissue tropism across organ systems in mammalian hosts (Silva Pereira et al. 2019). Taken together, the specificity of tropism for bartonellae and hemoplasmas in blood and blood‐related tissue may have contributed to our ability to detect more infections in vascular wing tissue compared to trypanosome infections. These limitations in infection detections suggest, again, that using wing tissues for pathogen detection may be best suited for pathogen discovery instead of general surveillance and estimating infection prevalence.

While blood‐borne pathogen detection in wing tissues may be limited, we quantified the sample sizes needed to obtain bartonellae, hemoplasma, or trypanosome infections for future pathogen discovery. Sample sizes needed to obtain a single infection and observed prevalence here were similar. With wing tissues in bats being collected and used for a variety of bat research topics, future collaborative sampling should contribute to better understanding the genetic diversity of these geographically widespread pathogens.

In conclusion, we show that using bat wing tissues for initial pathogen assessment and discovery is a complementary and less‐invasive sampling alternative to bat blood. Although detecting blood‐borne pathogens in wing tissues is likely not useful for estimating infection prevalence, sampling wild bat wing tissues for initial pathogen assessments and discovery can help guide more targeted and longitudinal surveillance efforts. We recommend bat biologists begin incorporating wing tissues into workflows for blood‐borne pathogen detection and discovery, including those vouchered and archived in museums. In doing so, using complementary wing tissues for blood‐borne pathogen detection will expand our fundamental knowledge of wildlife pathogen diversity and spillover risks.

## Author Contributions

D.J.B. conceived the idea. M.C.S., A.V.‐S. and D.J.B. designed methodology and carried out experiments. D.J.B., L.R.L., K.E.D. and K.E.S. collected field samples. N.B.S., M.B.F. and D.J.B. facilitated and coordinated all field work for sample collection. M.C.S., A.V.‐S., D.J.B., L.R.L., B.L.O., D.V.V. and K.E.S. all collected laboratory data. M.C.S. performed data analyses and led the writing of the manuscript. D.J.B. contributed editing to every manuscript version, and all other authors contributed critically to drafts and gave approval for final publication.

## Funding

This study was supported by the Explorers Club, National Institutes of Health (R03AI188200), Edward Mallinckrodt, Jr. Foundation, National Science Foundation (DBI 2515340, DEB 2508535), and Research Corporation for Science Advancement (RCSA), Subaward No. 28365, part of a USDA Non‐Assistance Cooperative Agreement with RCSA Federal Award No. 58‐3022‐0‐005. M.C.S. was supported by an appointment to the Intelligence Community Postdoctoral Research Fellowship Program at University of Oklahoma administered by Oak Ridge Institute for Science and Education through an interagency agreement between the U.S. Department of Energy and the Office of the Director of National Intelligence.

## Ethics Statement

Field procedures were performed according to guidelines for the safe and humane handling of bats published by the American Society of Mammalogists (Sikes and the Animal Care and Use Committee of the American Society of Mammalogists 2016) and were approved by the Institutional Animal Care and Use Committees of the American Museum of Natural History (AMNH IACUC‐20210614) and the University of Oklahoma (2022‐0197). All bat sampling was authorized by the Belize Forest Department through scientific collection permit FD/WL/1/21(12) and Belize Institute of Archaeology permits IA/S/5/6/2l(01) and IA/H/1/22(03).

## Conflicts of Interest

The authors declare no conflicts of interest.

## Supporting information


**Table S1:** PCR protocols and primers used for infection detection in both bat blood and wing tissues.

**Table S2:** Generalized linear models and outcomes for determining differences in infection positivity between wing tissue extraction methods using *Quick*‐DNA/RNA Viral Magbead Kit (control) compared to the *Quick*‐DNA Miniprep Plus Kit. All models were fit using a binomial response.

## Data Availability

Sequence data are available on GenBank under accessions PX794553, PX794555, PX794557‐76 (trypanosomes in blood); PV599394–96 (bartonellae in wing tissues); PX705789‐97 (hemoplasmas in wing tissues); and PX794554 and PX794556 (trypanosomes in wing tissues). Sequence data for bartonellae and hemoplasmas in blood have been provided previously (Lock et al. 2026). Positivity data and all R code are available via Zenodo (Simonis 2026).

## References

[mec70560-bib-0001] Álvarez‐Fernández, A. , E. B. Breitschwerdt , and L. Solano‐Gallego . 2018. “Bartonella Infections in Cats and Dogs Including Zoonotic Aspects.” Parasites and Vectors 11: 624. 10.1186/s13071-018-3152-6. PMC6280416 30514361

[mec70560-bib-0002] André, M. R. , C. H. Adania , S. M. Allegretti , and R. Z. Machado . 2011. “Hemoplasmas in Wild Canids and Felids in Brazil.” Journal of Zoo and Wildlife Medicine 42: 342–347. 10.1638/2010-0198.1. 22946419

[mec70560-bib-0003] Ansil, B. R. , A. Viswanathan , V. Ramachandran , H. M. Yeshwanth , A. Sanyal , and U. Ramakrishnan . 2025. “Host–Pathogen–Vector Continuum in a Changing Landscape: Potential Transmission Pathways for Bartonella in a Small Mammal Community.” Ecology and Evolution 15: e71085. 10.1002/ece3.71085. PMC11962204 40177689

[mec70560-bib-0004] Arnold, J. M. , S. A. Oswald , C. C. Voigt , et al. 2008. “Taking the Stress Out of Blood Collection: Comparison of Field Blood‐Sampling Techniques for Analysis of Baseline Corticosterone.” Journal of Avian Biology 39: 588–592. 10.1111/j.0908-8857.2008.04265.x.

[mec70560-bib-0005] Artois, M. , R. Bengis , R. J. Delahay , et al. 2009. “Wildlife Disease Surveillance and Monitoring.” In Management of Disease in Wild Mammals, edited by R. J. Delahay , G. C. Smith , and M. R. Hutchings , 187–213. Springer Japan. 10.1007/978-4-431-77134-0_10.

[mec70560-bib-0006] Bates, D. , M. Maechler , B. Bolker , and S. Walker . 2015. “Fitting Linear Mixed‐Effects Models Using lme4.” Journal of Statistical Software 67: 1–48.

[mec70560-bib-0007] Becker, D. J. , L. M. Bergner , A. B. Bentz , R. J. Orton , S. Altizer , and D. G. Streicker . 2018. “Genetic Diversity, Infection Prevalence, and Possible Transmission Routes of Bartonella spp. in Vampire Bats.” PLoS Neglected Tropical Diseases 12: e0006786. 10.1371/journal.pntd.0006786. PMC6159870 30260954

[mec70560-bib-0008] Becker, D. J. , K. E. Dyer , L. R. Lock , et al. 2024. “Geographically Widespread and Novel Hemotropic Mycoplasmas and Bartonellae in Mexican Free‐Tailed Bats and Sympatric North American Bat Species.” mSphere 10: e00116‐24. 10.1128/msphere.00116-24. PMC11774037 39660872

[mec70560-bib-0009] Becker, D. J. , K. E. Dyer , L. R. Lock , M. B. Fenton , and N. B. Simmons . 2025. “Habitat and Seasonal Drivers of Leukocyte Profiles Within and Across Neotropical Bat Species.” Biology Letters 21: 20250447. 10.1098/rsbl.2025.0447. PMC12570079 41159440

[mec70560-bib-0010] Becker, D. J. , K. A. Speer , A. M. Brown , et al. 2020. “Ecological and Evolutionary Drivers of Haemoplasma Infection and Bacterial Genotype Sharing in a Neotropical Bat Community.” Molecular Ecology 29: 1534–1549. 10.1111/mec.15422. PMC8299350 32243630

[mec70560-bib-0011] Becker, D. J. , K. A. Speer , J. M. Korstian , et al. 2021. “Disentangling Interactions Among Mercury, Immunity and Infection in a Neotropical Bat Community.” Journal of Applied Ecology 58: 879–889. 10.1111/1365-2664.13809. PMC8078557 33911313

[mec70560-bib-0012] Beckmann, S. , R. Freund , H. Pehl , A. Rodgers , and T. Venegas . 2019. “Rodent Species as Possible Reservoirs of *Borrelia burgdorferi* in a Prairie Ecosystem.” Ticks and Tick‐Borne Diseases 10: 1162–1167. 10.1016/j.ttbdis.2019.06.011. 31248821

[mec70560-bib-0013] Bergner, L. M. , D. J. Becker , C. Tello , J. E. Carrera , and D. G. Streicker . 2021. “Detection of *Trypanosoma cruzi* in the Saliva of Diverse Neotropical Bats.” Zoonoses and Public Health 68: 271–276. 10.1111/zph.12808. PMC8569697 33484236

[mec70560-bib-0014] Birtles, R. J. , and D. Raoult . 1996. “Comparison of Partial Citrate Synthase Gene (gltA) Sequences for Phylogenetic Analysis of Bartonella Species.” International Journal of Systematic Bacteriology 46: 891–897. 10.1099/00207713-46-4-891. 8863415

[mec70560-bib-0015] Boulouis, H.‐J. , C.‐C. Chang , J. Henn , R. Kasten , and B. Chomel . 2005. “Factors Associated With the Rapid Emergence of Zoonotic Bartonella Infections.” Veterinary Research 36: 383. 10.1051/vetres:2005009. 15845231

[mec70560-bib-0016] Breitschwerdt, E. B. 2008. “Feline Bartonellosis and Cat Scratch Disease.” Veterinary Immunology and Immunopathology 123: 167–171. 10.1016/j.vetimm.2008.01.025. 18295347

[mec70560-bib-0017] Champely, S. 2020. pwr: Basic Functions for Power Analysis.

[mec70560-bib-0018] Colwell, D. D. , F. Dantas‐Torres , and D. Otranto . 2011. “Vector‐Borne Parasitic Zoonoses: Emerging Scenarios and New Perspectives.” Veterinary Parasitology 182: 14–21. 10.1016/j.vetpar.2011.07.012. 21852040

[mec70560-bib-0019] Cronin, C. E. , M. M. Juman , A. J. Richardson , C. A. Whittier , D. J. Becker , and A. W. Ferguson . 2025. Biodiversity Databases as Underutilized Resources for Pathogen Discovery: A Quantitative Synthesis of Bat and Rodent Tissue Collections in Natural History Museums. bioRxiv. 10.1101/2025.11.28.691153.

[mec70560-bib-0020] Daszak, P. 2000. “Emerging Infectious Diseases of Wildlife — Threats to Biodiversity and Human Health.” Science 287: 443–449. 10.1126/science.287.5452.443. 10642539

[mec70560-bib-0021] de Noya, B. A. , and O. N. González . 2015. “An Ecological Overview on the Factors That Drives to *Trypanosoma cruzi* Oral Transmission.” Acta Tropica 151: 94–102. 10.1016/j.actatropica.2015.06.004. 26066984

[mec70560-bib-0022] Dehio, C. 2004. “Molecular and Cellular Basis of Bartonella Pathogenesis.” Annual Review of Microbiology 58: 365–390. 10.1146/annurev.micro.58.030603.123700. 15487942

[mec70560-bib-0023] Delgado, M. , C. Wannous , P. Tizzani , et al. 2023. “Wildlife Health Surveillance: Gaps, Needs and Opportunities.” Revue Scientifique et Technique 42: 161–172. 10.20506/rst.42.3359. 37232308

[mec70560-bib-0024] Descloux, E. , O. Mediannikov , A. C. Gourinat , et al. 2021. “Flying Fox Hemolytic Fever, Description of a New Zoonosis Caused by Candidatus Mycoplasma Haemohominis.” Clinical Infectious Diseases 73: e1445–e1453. 10.1093/cid/ciaa1648. 33119064

[mec70560-bib-0025] Desquesnes, M. , A. Dargantes , D.‐H. Lai , Z.‐R. Lun , P. Holzmuller , and S. Jittapalapong . 2013. “Trypanosoma Evansi and Surra: A Review and Perspectives on Transmission, Epidemiology and Control, Impact, and Zoonotic Aspects.” BioMed Research International 2013: 321237. 10.1155/2013/321237. PMC3789323 24151595

[mec70560-bib-0087] Dunsmore, A. , K. E. Dyer , L. R. Lock , et al. 2025. “Vampire Bats in Belize Harbor Multiple *Trypanosoma cruzi* Genotypes: Implications for Parasite Transmission at the Wildlife–Domestic–Human Interface.” PLoS Neglected Tropical Diseases 20: e0013852. 10.1371/journal.pntd.0013852. PMC13592685 42726894

[mec70560-bib-0027] Eicher, S. C. , and C. Dehio . 2012. “Bartonella Entry Mechanisms Into Mammalian Host Cells.” Cellular Microbiology 14: 1166–1173. 10.1111/j.1462-5822.2012.01806.x. 22519749

[mec70560-bib-0028] Fisher, T. A. , S. M. Coker , R. Francisco , et al. 2025. “Prevalence of Trypanosoma cruzi Antibodies in Wild Pigs ( *Sus scrofa* ) in the USA.” Journal of Wildlife Diseases 61: 1071–1076. 10.7589/JWD-D-25-00020. 40965471

[mec70560-bib-0029] Fox, J. , and S. Weisberg . 2019. An R Companion to Applied Regression. Sage. Third ed. https://socialsciences.mcmaster.ca/jfox/Books/Companion/.

[mec70560-bib-0030] Grimshaw, J. R. , D. Donner , R. Perry , et al. 2024. “Disentangling Genetic Diversity of *Myotis septentrionalis* : Population Structure, Demographic History, and Effective Population Size.” Journal of Mammalogy 105: 854–864. 10.1093/jmammal/gyae056.

[mec70560-bib-0031] Haghani, A. , C. Z. Li , T. R. Robeck , et al. 2023. “DNA Methylation Networks Underlying Mammalian Traits.” Science 381: eabq5693. 10.1126/science.abq5693. PMC11180965 37561875

[mec70560-bib-0032] Herrera, J. P. , N. Duncan , E. Clare , M. B. Fenton , and N. Simmons . 2018. “Disassembly of Fragmented Bat Communities in Orange Walk District, Belize.” Acta Chiropterologica 20: 147–159. 10.3161/15081109ACC2018.20.1.011.

[mec70560-bib-0033] Ingala, M. R. , D. J. Becker , J. Bak Holm , K. Kristiansen , and N. B. Simmons . 2019. “Habitat Fragmentation Is Associated With Dietary Shifts and Microbiota Variability in Common Vampire Bats.” Ecology and Evolution 9: 6508–6523. 10.1002/ece3.5228. PMC6580296 31236240

[mec70560-bib-0034] Jones, K. E. , N. G. Patel , M. A. Levy , et al. 2008. “Global Trends in Emerging Infectious Diseases.” Nature 44: 319–335. 10.1038/nature06536. PMC5960580 18288193

[mec70560-bib-0035] Kang, J.‐G. , H.‐C. Kim , C.‐Y. Choi , et al. 2013. “Molecular Detection of Anaplasma, Bartonella, and Borrelia Species in Ticks Collected From Migratory Birds From Hong‐Do Island, Republic of Korea.” Vector Borne and Zoonotic Diseases 13: 215–225. 10.1089/vbz.2012.1149. 23428091

[mec70560-bib-0036] Kearse, M. , R. Moir , A. Wilson , et al. 2012. “Geneious Basic: An Integrated and Extendable Desktop Software Platform for the Organization and Analysis of Sequence Data.” Bioinformatics 28: 1647–1649. 10.1093/bioinformatics/bts199. PMC3371832 22543367

[mec70560-bib-0037] Klement‐Frutos, E. , O. Mediannikov , P.‐E. Fournier , J. Haroche , V. Leblond , and E. Caumes . 2023. “Mycoplasma Haemohominis as a Cause of Fever of Unknown Origin in a Traveller.” Journal of Travel Medicine 30: taac129. 10.1093/jtm/taac129. 36331263

[mec70560-bib-0038] Kruskop, S. V. , A. V. Borisenko , N. V. Ivanova , B. K. Lim , and J. L. Eger . 2012. “Genetic Diversity of Northeastern Palaearctic Bats as Revealed by DNA Barcodes.” Acta Chiropterologica 14: 1–14. 10.3161/150811012X654222.

[mec70560-bib-0039] Lenth, R. 2021. emmeans: Estimated Marginal Means, aka Least‐Squares Means.

[mec70560-bib-0040] Lilley, T. M. , C. A. Wilson , R. F. Bernard , et al. 2017. “Molecular Detection of Candidatus Bartonella Mayotimonensis in North American Bats.” Vector‐Borne and Zoonotic Diseases 17: 243–246. 10.1089/vbz.2016.2080. 28165925

[mec70560-bib-0041] Lima, M. A. , M. E. Vaz , J. E. Ferreira , et al. 2025. “Hidden Diversity of Trypanosomatidae (Protozoa: Kinetoplastea) in Bats From an Urban Park in Brazil.” PLoS One 20: e0319781. 10.1371/journal.pone.0319781. PMC11913285 40096043

[mec70560-bib-0086] Lock, L. R. , K. E. Dyer , D. V. Volokhov , et al. 2026. “Longitudinal Impacts of Forest Loss on Bartonella and Hemotropic Mycoplasma Dynamics in Vampire Bat Within a Fragmented Habitat.” Molecular Ecology 35: e70466. 10.1111/mec.70466. PMC13355873 42435377

[mec70560-bib-0043] Lourenço, J. L. M. , T. T. C. Minuzzi‐Souza , L. R. Silva , et al. 2018. “High Frequency of Trypanosomatids in Gallery Forest Bats of a Neotropical Savanna.” Acta Tropica 177: 200–206. 10.1016/j.actatropica.2017.10.012. 29050949

[mec70560-bib-0044] Lu, A. T. , Z. Fei , A. Haghani , et al. 2023. “Universal DNA Methylation Age Across Mammalian Tissues.” Nat Aging 3: 1144–1166. 10.1038/s43587-023-00462-6. PMC10501909 37563227

[mec70560-bib-0045] Manvell, C. , K. Ferris , R. Maggi , E. B. Breitschwerdt , and E. Lashnits . 2021. “Prevalence of Vector‐Borne Pathogens in Reproductive and Non‐Reproductive Tissue Samples From Free‐Roaming Domestic Cats in the South Atlantic USA.” Pathogens 10: 1221. 10.3390/pathogens10091221. PMC8472639 34578253

[mec70560-bib-0046] Martínez‐Hernández, F. , B. Oria‐Martínez , E. Rendón‐Franco , G. Villalobos , and C. I. Muñoz‐García . 2022. “ *Trypanosoma cruzi*, Beyond the Dogma of Non‐Infection in Birds.” Infection, Genetics and Evolution 99: 105239. 10.1016/j.meegid.2022.105239. 35144004

[mec70560-bib-0085] McKee, C. D. , C. T. Webb , M. Y. Kosoy , et al. 2026. “Manipulating Vector Transmission Reveals Local Processes in Bartonella Communities of Bats.” Parasitology 153: 420–431. 10.1017/S0031182026101656. PMC13215733 41622806

[mec70560-bib-0048] Medellín, R. A. , H. T. Arita , and Ó. Sánchez . 2007. Bats of Mexico: Identification Field Key. 2nd ed. National Autonomous University of Mexico, Mexico City, Mexico.

[mec70560-bib-0049] Messick, J. B. 2004. “Hemotrophic Mycoplasmas (Hemoplasmas): A Review and New Insights Into Pathogenic Potential.” Veterinary Clinical Pathology 33: 2–13. 10.1111/j.1939-165X.2004.tb00342.x. 15048620

[mec70560-bib-0050] Millán, J. , S. Di Cataldo , D. V. Volokhov , and D. J. Becker . 2021. “Worldwide Occurrence of Haemoplasmas in Wildlife: Insights Into the Patterns of Infection, Transmission, Pathology and Zoonotic Potential.” Transboundary and Emerging Diseases 68: 3236–3256. 10.1111/tbed.13932. 33210822

[mec70560-bib-0051] Nichols, M. D. , W. D. Lord , and W. L. Haynie . 2019. “ *Trypanosoma cruzi* in a Mexican Free‐Tailed Bat ( *Tadarida brasiliensis* ) in Oklahoma, USA.” Journal of Wildlife Diseases 55: 444. 10.7589/2018-04-095. 30277832

[mec70560-bib-0052] Norman, A. F. , R. Regnery , P. Jameson , C. Greene , and D. C. Krause . 1995. “Differentiation of Bartonella‐Like Isolates at the Species Level by PCR‐Restriction Fragment Length Polymorphism in the Citrate Synthase Gene.” Journal of Clinical Microbiology 33: 1797–1803. 10.1128/jcm.33.7.1797-1803.1995. PMC228273 7545181

[mec70560-bib-0053] Noyes, H. a. , J. R. Stevens , M. Teixeira , J. Phelan , and P. Holz . 1999. “A Nested PCR for the ssrRNA Gene Detects Trypanosoma Binneyi in the Platypus and Trypanosoma sp. in Wombats and Kangaroos in Australia1.” International Journal for Parasitology 29: 331–339. 10.1016/S0020-7519(98)00167-2. 10221634

[mec70560-bib-0054] Oberpriller, J. , M. de Souza Leite , and M. Pichler . 2022. “Fixed or Random? On the Reliability of Mixed‐Effects Models for a Small Number of Levels in Grouping Variables.” Ecology and Evolution 12: e9062. 10.1002/ece3.9062. PMC9309037 35898418

[mec70560-bib-0055] Pavlova, E. V. , G. S. Alekseeva , M. N. Erofeeva , N. A. Vasilieva , A. V. Tchabovsky , and S. V. Naidenko . 2018. “The Method Matters: The Effect of Handling Time on Cortisol Level and Blood Parameters in Wild Cats.” Journal of Experimental Zoology Part A: Ecological and Integrative Physiology 329: 112–119. 10.1002/jez.2191. 29893473

[mec70560-bib-0056] Quinet, C. , G. Czaplicki , E. Dion , F. D. Pozzo , A. Kurz , and C. Saegerman . 2016. “First Results in the Use of Bovine Ear Notch Tag for Bovine Viral Diarrhoea Virus Detection and Genetic Analysis.” PLoS One 11: e0164451. 10.1371/journal.pone.0164451. PMC5072587 27764130

[mec70560-bib-0057] Quiroga, N. , R. Campos‐Soto , A. Yañez‐Meza , et al. 2022. “ *Trypanosoma cruzi* DNA in *Desmodus rotundus* (Common Vampire Bat) and *Histiotus montanus* (Small Big‐Eared Brown Bat) From Chile.” Acta Tropica 225: 106206. 10.1016/j.actatropica.2021.106206. 34687642

[mec70560-bib-0058] R Core Team . 2023. R: A language and Environment for Statistical Computing.

[mec70560-bib-0084] Ramírez, J. D. , C. Hernández , M. Montilla , et al. 2014. “First Report of Human *Trypanosoma cruzi* Infection Attributed to TcBat Genotype.” Zoonoses and Public Health 61: 477–479. 10.1111/zph.12094. 25285940

[mec70560-bib-0060] Reid, F. A. 2009. A Field Guide to the Mammals of Central America and Southeast Mexico. 2nd ed. Oxford University Press.

[mec70560-bib-0061] Roswag, A. , N. I. Becker , and J. A. Encarnação . 2014. “Factors Influencing Stable Nitrogen Isotope Ratios in Wing Membranes of Insectivorous Bat Species: A Field Study.” Mammalian Biology 79: 110–116. 10.1016/j.mambio.2013.10.006.

[mec70560-bib-0062] Ryser‐Degiorgis, M.‐P. 2013. “Wildlife Health Investigations: Needs, Challenges and Recommendations.” BMC Veterinary Research 9: 223. 10.1186/1746-6148-9-223. PMC4228302 24188616

[mec70560-bib-0063] Sharan, M. , D. Vijay , J. P. Yadav , J. S. Bedi , and P. Dhaka . 2023. “Surveillance and Response Strategies for Zoonotic Diseases: A Comprehensive Review.” Science in One Health 2: 100050. 10.1016/j.soh.2023.100050. PMC11262259 39077041

[mec70560-bib-0064] Sikes, R. S. , and the Animal Care and Use Committee of the American Society of Mammalogists . 2016. “2016 Guidelines of the American Society of Mammalogists for the Use of Wild Mammals in Research and Education.” Journal of Mammalogy 97: 663–688. 10.1093/jmammal/gyw078. PMC5909806 29692469

[mec70560-bib-0065] Silva Pereira, S. , S. Trindade , M. De Niz , and L. M. Figueiredo . 2019. “Tissue Tropism in Parasitic Diseases.” Open Biology 9: 190036. 10.1098/rsob.190036. PMC6544988 31088251

[mec70560-bib-0083] Simonis, M. C. 2026. “Simonimc/Validating‐Wing‐Biopsies‐for‐Blood‐Borne‐Pathogen‐Characterization‐in‐Bats: Validating Wing Biopsies for Blood‐Borne Pathogen Characterization in Bats.” Zenodo. 10.5281/zenodo.18947640.

[mec70560-bib-0067] Sinsky, R. J. , and J. Piesman . 1989. “Ear Punch Biopsy Method for Detection and Isolation of *Borrelia burgdorferi* From Rodents.” Journal of Clinical Microbiology 27: 1723–1727. 10.1128/jcm.27.8.1723-1727.1989. PMC267661 2768461

[mec70560-bib-0068] Szentivanyi, T. , C. McKee , G. Jones , and J. T. Foster . 2023. “Trends in Bacterial Pathogens of Bats: Global Distribution and Knowledge Gaps.” Transboundary and Emerging Diseases 2023: e9285855. 10.1155/2023/9285855. PMC12017137 40303798

[mec70560-bib-0069] Thompson, C. W. , K. L. Phelps , M. W. Allard , et al. 2021. “Preserve a Voucher Specimen! The Critical Need for Integrating Natural History Collections in Infectious Disease Studies.” MBio 12. 10.1128/mbio.02698-20. PMC7844540 33436435

[mec70560-bib-0082] Torgerson, E. G. , M. Adams , L. R. Lock , et al. 2026. “Neotropical Bats as Sentinels for Emerging Zoonoses in Central America: A Case Study Identifying *Trypanosoma cruzi* in Bats From Belize Using Metagenomic Next‐Generation Sequencing.” PLoS Neglected Tropical Diseases 20: e0013851. 10.1371/journal.pntd.0013851. PMC13395375 42490589

[mec70560-bib-0071] Trüeb, I. , R. D. Portela , C. R. Franke , et al. 2018. “ *Trypanosoma cruzi* and *Leishmania* SP. Infection in Wildlife From Urban Rainforest Fragments in Northeast Brazil.” Journal of Wildlife Diseases 54: 76. 10.7589/2017-01-017. 28977769

[mec70560-bib-0072] Vicente‐Santos, A. , L. R. Lock , M. Allira , et al. 2023. “Serum Proteomics Reveals a Tolerant Immune Phenotype Across Multiple Pathogen Taxa in Wild Vampire Bats.” Frontiers in Immunology 14: 1281732. 10.3389/fimmu.2023.1281732. PMC10773587 38193073

[mec70560-bib-0073] Volokhov, D. V. , D. J. Becker , L. M. Bergner , et al. 2017. “Novel Hemotropic Mycoplasmas Are Widespread and Genetically Diverse in Vampire Bats.” Epidemiology and Infection 145: 3154–3167. 10.1017/S095026881700231X. PMC6538534 29061202

[mec70560-bib-0074] Volokhov, D. V. , L. R. Lock , K. E. Dyer , et al. 2023. “Expanded Diversity of Novel Hemoplasmas in Rare and Undersampled Neotropical Bats.” One Health 17: 100633. 10.1016/j.onehlt.2023.100633. PMC10618802 37920218

[mec70560-bib-0075] Walker, F. M. , C. H. D. Williamson , D. E. Sanchez , C. J. Sobek , and C. L. Chambers . 2016. “Species From Feces: Order‐Wide Identification of Chiroptera From Guano and Other Non‐Invasive Genetic Samples.” PLoS One 11: e0162342. 10.1371/journal.pone.0162342. PMC5031397 27654850

[mec70560-bib-0076] Watsa, M. , and Wildlife Disease Surveillance Focus Group . 2020. “Rigorous Wildlife Disease Surveillance.” Science 369: 145–147. 10.1126/science.abc0017. 32646989

[mec70560-bib-0077] Wickham, H. 2016. ggplot2: Elegant Graphics for Data Analysis. Springer‐Verlag.

[mec70560-bib-0078] Widmaier, E. P. , and T. H. Kunz . 1993. “Basal, Diurnal, and Stress‐Induced Levels of Glucose and Glucocorticoids in Captive Bats.” Journal of Experimental Zoology 265: 533–540. 10.1002/jez.1402650509. 8468542

[mec70560-bib-0079] Wilkinson, G. S. , D. M. Adams , A. Haghani , et al. 2021. “DNA Methylation Predicts Age and Provides Insight Into Exceptional Longevity of Bats.” Nature Communications 12: 1615. 10.1038/s41467-021-21900-2. PMC7955057 33712580

[mec70560-bib-0080] Willi, B. , F. S. Boretti , S. Tasker , et al. 2007. “From *Haemobartonella* to Hemoplasma: Molecular Methods Provide New Insights.” Veterinary Microbiology 125: 197–209. 10.1016/j.vetmic.2007.06.027. 17706380

[mec70560-bib-0081] York, H. A. , B. Rodríguez‐Herrera , R. K. Laval , R. M. Timm , and K. E. Lindsay . 2019. “Field Key to the Bats of Costa Rica and Nicaragua.” Journal of Mammalogy 100: 1726–1749. 10.1093/jmammal/gyz150.

